# Molecular profiling of ALDH1^+^ colorectal cancer stem cells reveals preferential activation of MAPK, FAK, and oxidative stress pro-survival signalling pathways

**DOI:** 10.18632/oncotarget.24420

**Published:** 2018-02-05

**Authors:** Radhakrishnan Vishnubalaji, Muthurangan Manikandan, Mohamed Fahad, Rimi Hamam, Musaad Alfayez, Moustapha Kassem, Abdullah Aldahmash, Nehad M. Alajez

**Affiliations:** ^1^ Stem Cell Unit, Department of Anatomy, College of Medicine, King Saud University, Riyadh, Saudi Arabia; ^2^ Molecular Endocrinology Unit (KMEB), Department of Endocrinology, University Hospital of Odense and University of Southern Denmark, Odense, Denmark; ^3^ Departement de Medecine, Universite de Montreal, Montreal, Canada; ^4^ Department of Cellular and Molecular Medicine, Danish Stem Cell Center (DanStem), University of Copenhagen, Copenhagen, Denmark; ^5^ Prince Naif Health Research Center, King Saud University, Riyadh, Saudi Arabia

**Keywords:** colorectal cancer, cancer stem cells, ALDH, microarray, resistance

## Abstract

Tumour heterogeneity leads to variable clinical response and inaccurate diagnostic and prognostic assessment. Cancer stem cells (CSCs) represent a subpopulation responsible for invasion, metastasis, therapeutic resistance, and recurrence in many human cancer types. However, the true identity of colorectal cancer (CRC) SCs remains elusive. Here, we aimed to characterize and define the gene expression portrait of CSCs in CRC-model SW403 cells. We found that ALDH^+^ positive cells are clonogenic and highly proliferative; their global gene expression profiling-based molecular signature revealed gene enrichment related to DNA damage, MAPK, FAK, oxidative stress response, and Wnt signalling. ALDH^+^ cells showed enhanced ROS stress resistance, whereas MAPK/FAK pathway pharmacologic inhibition limited their survival. Conversely, 5-fluorouracil increased the ALDH^+^ cell fraction among the SW403, HCT116 and SW620 CRC models. Notably, analysis of *ALDH1A1* and POU5F1 expression levels in cohorts of 462 or 420 patients for overall (OS) or disease-free (DFS) survival, respectively, obtained from the Cancer Genome Atlas CRC dataset, revealed strong association between elevated expression and poor OS (*p* = 0.006) and poor DFS (*p* = 0.05), thus implicating *ALDH1A1* and POU5F1 in CRC prognosis. Our data reveal distinct molecular signature of ALDH^+^ CSCs in CRC and suggest pathways relevant for successful targeted therapies and management of CRC.

## INTRODUCTION

Cancer represents the second leading cause of morbidity worldwide. GLOBOCAN 2012 estimated 14.1 million new cancer cases and 8.2 million cancer deaths occurred in 2012 across the globe. Among these, colorectal cancer (CRC) comprises the third most common cancer with 1.4 million new cases and was responsible for 693,900 deaths in 2012, with higher mortality rate in males compared to females [1, 2]. The most common treatment for localized CRC is surgical removal; however, patients with CRC often presented with metastatic disease or exhibit high probability of developing disseminated disease during their lifetime [3]. Consistent with this, the leading cause of CRC mortality is the failure of most therapies in patients with metastatic disease. To reduce mortality from CRC, it is therefore important to develop novel approaches based on the cellular and molecular phenotyping of CRC. Notably, our previous study has revealed multiple deregulated signalling pathways in CRC and suggested targeting those networks as a potential therapeutic strategy for CRC [4].

Treatment choices for patients with CRC assume homogeneity in tumour mass; therefore it is plausible that conventional chemotherapy and radiotherapy sometimes fail in tumour eradication. Alternatively, several studies have demonstrated that solid tumours including CRC exhibit cellular heterogeneity [5, 6], which may reflect different origin of cells at the time of tumour origination [7]. Furthermore, recently the presence within solid tumours of a minor subset of cells termed cancer stem-like cells (CSCs) or tumour-initiating cells (TICs), which exhibit self-renewal and differentiation capabilities, has been demonstrated and suggested to be responsible for tumour maintenance, metastasis and drug resistance [6, 8, 9]. Although normal tissue stem cells (SCs) and CSCs share certain characteristics, they exhibit significant differences in their differentiation potential and their microenvironmental niches [10, 11].

A number of surface markers e.g. CD133 (prominin-1), CD44, and CD29 have been reported as potential markers for different types of CSCs [8]; however, these CD antigens also exist on normal stem cells and thus are of low specificity [12, 13]. In particular, aldehyde dehydrogenase 1 (ALDH1) has been reported as a marker for CSCs in a number of cancers [14, 15]. While there are three different isoforms for ALDH1 (ALDH1A1, ALDH1A2, and ALDH1A3), ALDH1 activity is predominantly attributed to ALDH1A1 isotype [11]. ALDH1A1-expressing CSCs from breast, lung, head and neck squamous cancer, possess tumour-initiating capabilities, suggesting a role in supporting tumour proliferation and maintenance [16–18]. The relevance of CSCs in cancer has been demonstrated in studies aimed at targeting CSC subpopulations and their signalling pathways [6, 9, 19]. In one study, patients with rectal adenocarcinoma (*n* = 64) who received preoperative radiochemotherapy showed high expression levels of different CSC markers—CD44, LGR5, CD166, and ALDH1—by immunostaining; additionally, in a Cox proportional hazards multiple regression model, ALDH1 independently predicted poor prognosis in patients with CRC who received radiochemotherapy [20].

Although CSCs have been identified in many different types of solid tumours, the identity of ALDH^+^ CSCs and their molecular signature as well as their clonogenic and drug resistance properties are poorly characterized. In the current study, we utilized fluorescence activated cell sorting (FACS) and isolated the ALDH1^+^ and ALDH1^−^ populations from the SW403 CRC cell model, characterised their molecular and functional phenotype, and subsequently validated these in additional CRC cell models. Our data identified several preferentially activated signalling pathways in ALDH1^+^ cells related to drug resistance with potential therapeutic implications that correlated with CRC prognosis.

## RESULTS

### Functional and molecular characterisation of an ALDH^+^ population in CRC SW403 cells.

**Figure 1:**
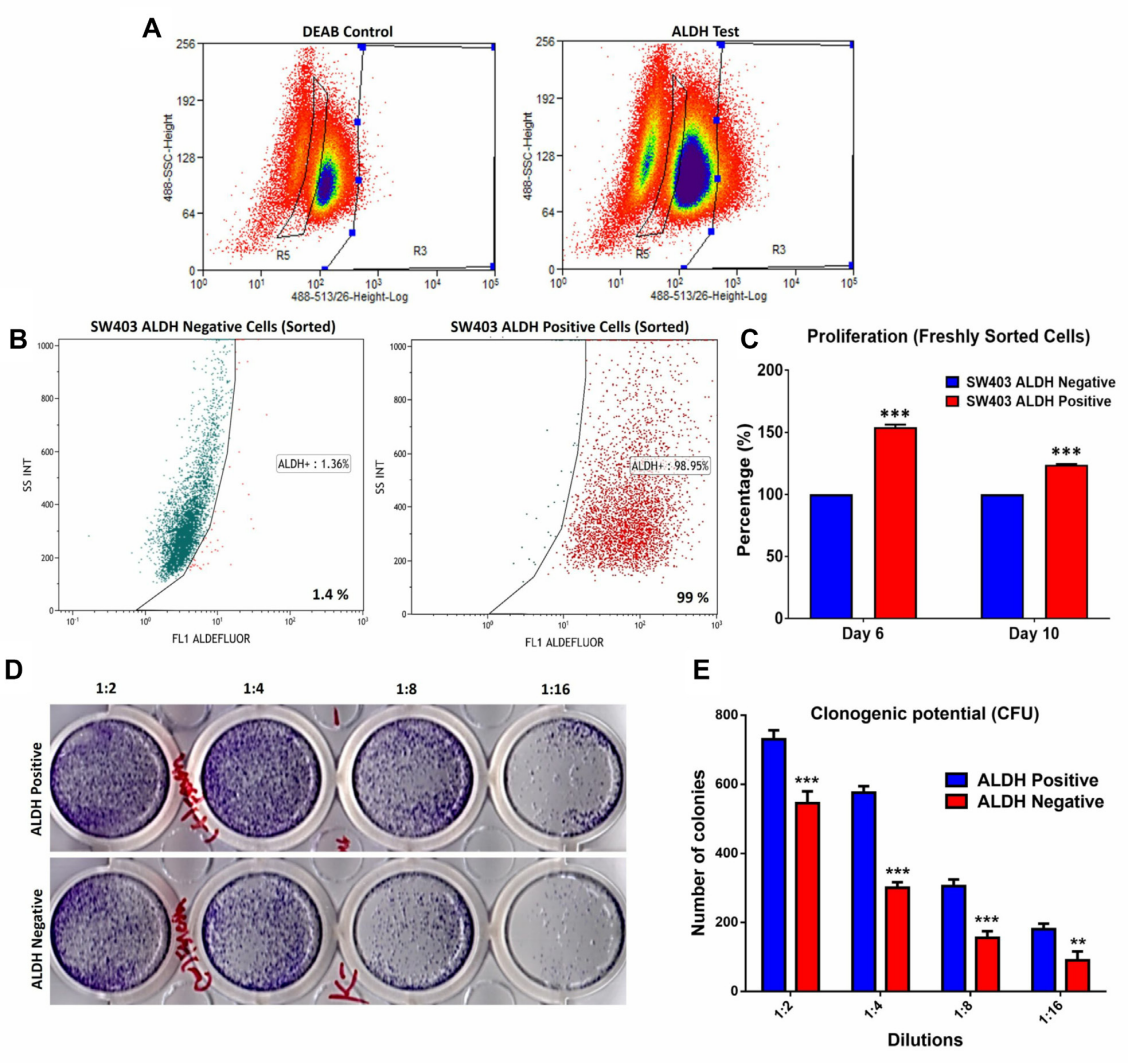
Proliferation and clonogenic potential of colorectal cancer ALDH^+^ cells (**A**) Frequency of ALDH^+^ cells in the SW403 CRC model measured using the Aldefluor^®^ assay and flow cytometry. The shift of fluorescence defined the population in R3 (Right panel) presenting positive ALDH1 activity and in R5 presenting Negative ALDH1 activity. The highly positive sub-population (∼5%) and ALDH^−^ cells were collected using the Astrios^®^ cell sorter. (**B**) Purity assessment was performed on sorted ALDH^+^ positive and negative subpopulations using the Aldefluor assay where the percentage of ALDH^+^ was ∼99% compared to the negative fraction ∼1.4%. (**C**) Proliferation of ALDH^+^ positive cells compared to ALDH^−^ cells over time. (**D** and **E**) Clonogenic assay showing marked increase in the colony forming capability of ALDH^+^ compared to ALDH^−^ cells. Plates were stained with Diff-Quik stain set on day 10. Wells are representative of two independent experiments for each condition. (e) The two-tailed *t*-test was used to compare different groups. ^**^p < 0.005; ^***^p < 0.0005.

We employed the SW403 cell line as a cell model for CRC and assessed the expression of several colorectal CSC-associated markers [8] (Supplementary Figure 1). The cells exhibited heterogeneous expression of ALDH (7%), LGR5 (4%), and CD90 (3%), whereas CD133 (99%), EpCAM (100%), CD44 (100%), and CD29 (100%) were expressed by the whole cell population. The SW403 cells were CD24^−^ (0%). The frequency of the ALDH^+^ population in SW403 cells was determined using an Aldefluor assay. As shown in Figure 1A, approximately 7% of the cells were ALDH^+^, which decreased to <1% in the presence of diethylaminobenzaldehyde (DEAB) (an ALDH inhibitor). Subsequently, we sorted both ALDH^+^ and ALDH^−^ cell fractions using FACS. The purity of sorted cells was analysed by Aldefluor assay, which revealed more than 99% purity in the ALDH^+^ fraction, whereas the sorted ALDH^−^ fraction showed minimal ALDH activity of <1.4% (Figure 1B). Further analysis demonstrated that the percentage of proliferating cells was higher in ALDH^+^ cells (day 6: 154% *vs* 100% and day 10: 124% *vs* 100%) compared to ALDH^−^ cells, *p* < 0.0005 (Figure 1C). This decrease in relative proliferation rate between day 10 and day 6 could possibly be attributed to the re-expression of ALDH by ALDH- fraction in culture (Supplementary Figure 2). Concordantly, the number of colonies formed in the ALDH^+^ fraction was higher than that observed in the ALDH^–^ fraction (Figure 1D and 1E). Taken together, our data demonstrated higher proliferation and clonogenic capability of the ALDH^+^ cells. We observed significant increase in a number of stem cell associated gene markers: *KL4*, *BAX*, *PCNA*, *SMOC2*, *KITLG*, *NANOG*, *KLF5*, and *BST* and decrease in the expression of *CD133*, *CDH1*, *SOX4*, and *SOX2* in ALDH^+^ compared to ALDH^−^ cells (Supplementary Figure 3). The expression of *PLAU*, *SNAI1*, *BMI1*, and *LGR5* did not show significant change in ALDH^+^ compared to ALDH^−^ cells.

### Global gene expression profiling reveals a distinct molecular profile of ALDH^+^ cells

**Figure 2:**
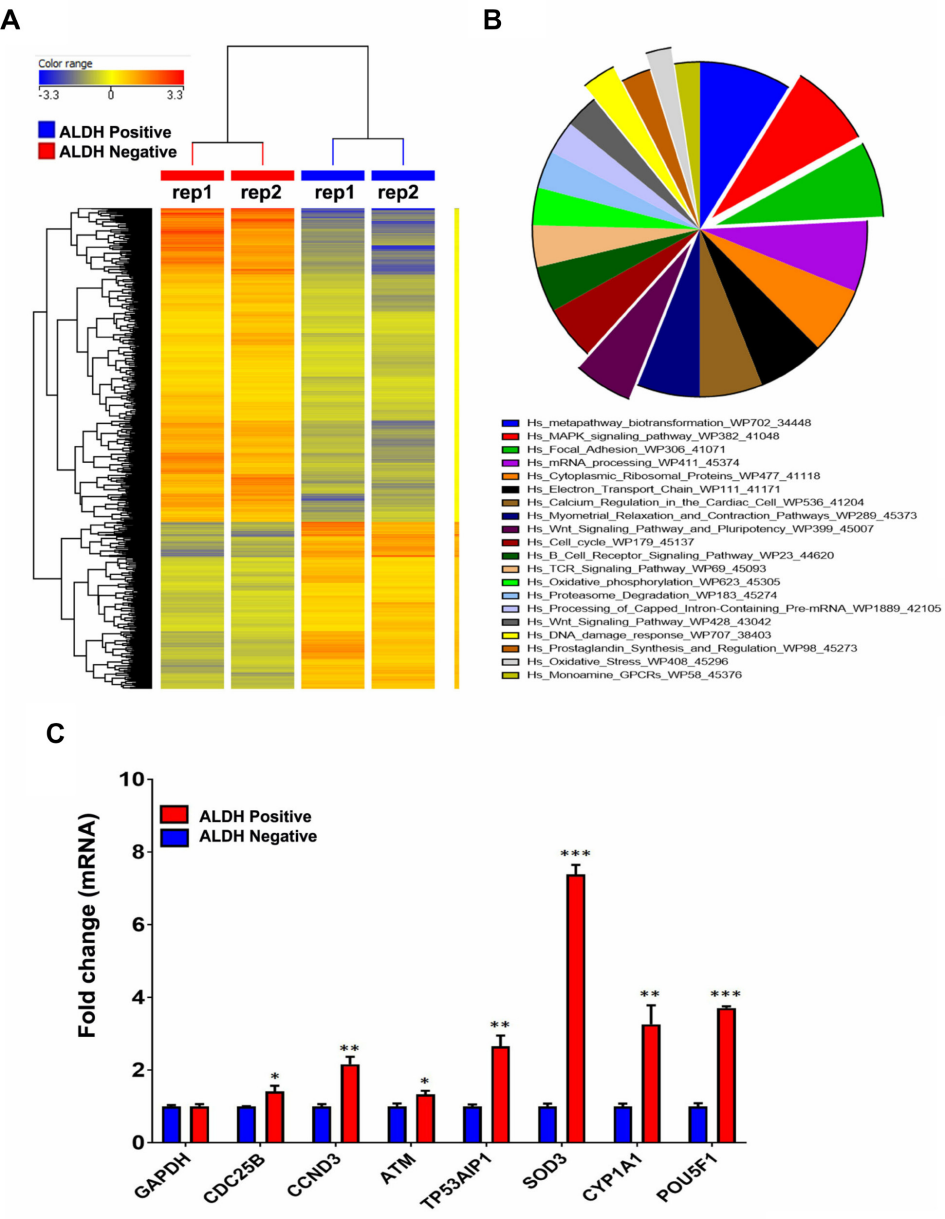
Preferential activation of multiple signalling pathways in ALDH^+^ cells (**A**) Hierarchical clustering of ALDH^+^ vs ALDH^−^ subpopulations based on differentially expressed mRNA levels. Each column represents one replica and each row represents a transcript. Expression level of each gene in a single sample is depicted according to the colour scale. (**B**) Pie chart illustrating the distribution of the top 20 pathways designations for the differentially expressed genes in ALDH^+^ cells. The pie size corresponds to the number of matched entities. (**C**) Expression levels of selected genes from the microarray data were validated using qRT-PCR in ALDH1^+^ compared to ALDH^−^ cells. Data are presented as the means ± S.D, *n* = 3. ^*^p < 0.05; ^**^p < 0.005; ^***^p < 0.0005.

We subsequently performed global mRNA expression profiling comparing ALDH^+^ to ALDH^−^ cells. As shown in Figure 2A, hierarchical clustering based on differentially expressed mRNAs revealed clear separation of ALDH^+^ from ALDH^−^ cells. We observed 1015 up-regulated and 1906 downregulated transcripts in ALDH^+^ cells compared to ALDH^−^ cells (Supplementary Table 1). The distribution of the top 20 enriched pathways for the up-regulated genes in ALDH^+^ cells is shown in Figure 2B. Among the highly enriched pathways were: DNA damage and oxidative stress response, MAPK, FAK, and Wnt signalling, and pluripotency. The expression levels of a selected group of genes related to Cell cycle, DNA damage, oxidative stress, Wnt and apoptosis pathways including *CDC25B*, *CCND3*, *ATM*, *TP53AIP1*, *SOD3*, *CYP1A1*, and *POU5F1* were validated using quantitative reverse transcription polymerase chain reaction (qRT-PCR) analysis (Figure 2C).

### ALDH^+^ cells exhibit enhanced resistance to 5-Fluorouracil (5-FU) drug treatment

**Figure 3:**
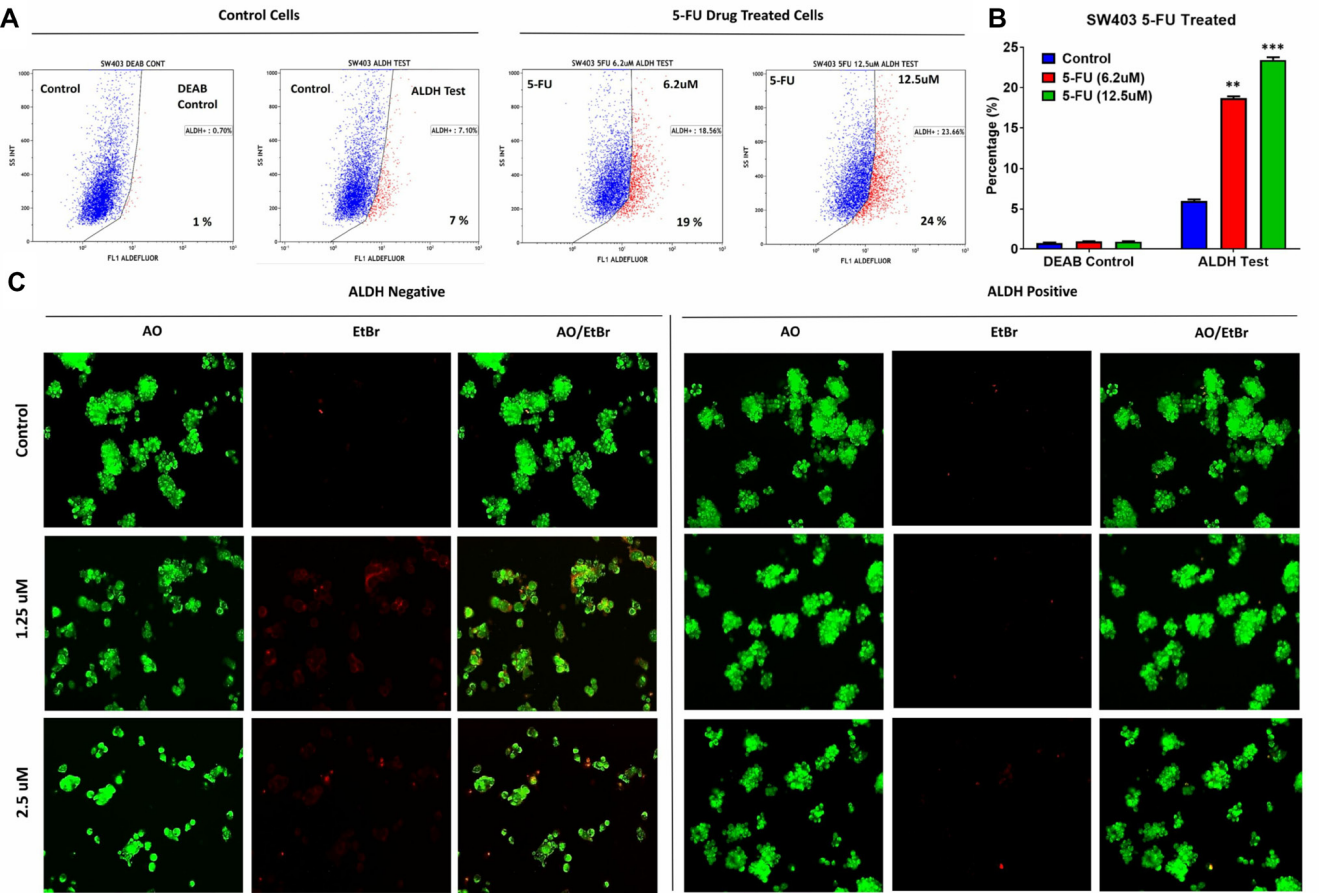
ALDH^+^ cells show enhanced resistance to 5-fluorouracil (**A**) Unsorted SW403 cells were exposed to 5-FU for 5 days, and subsequently the frequencies of ALDH^+^ cells were assessed using the Aldefluor assay, which revealed a dose-dependent increase in the proportion of ALDH^+^ cells (Control (7%), 5-Fu treated at 6.2 μM (19%), 12.5 μM (24%), and 25 μM (22%)), respectively. Experiments were normalized to the respective DEAB controls. (**B**) Quantification of the percentages of ALDH^+^ cells from (a). The two-tailed *t*-test was used to compare different treatment groups. ^**^p < 0.005.; ^***^p < 0.0005. (**C**) Representative fluorescence images of sorted ALDH^+^ and ALDH^−^ subpopulations [± different concentration (1.25 μM and 2.5 μM) 5-fluorouracil]. Cells were stained with acridine orange/ethidium bromide to detect apoptotic (cells with green condensed chromatin) and necrotic cells (red).

**Figure 4:**
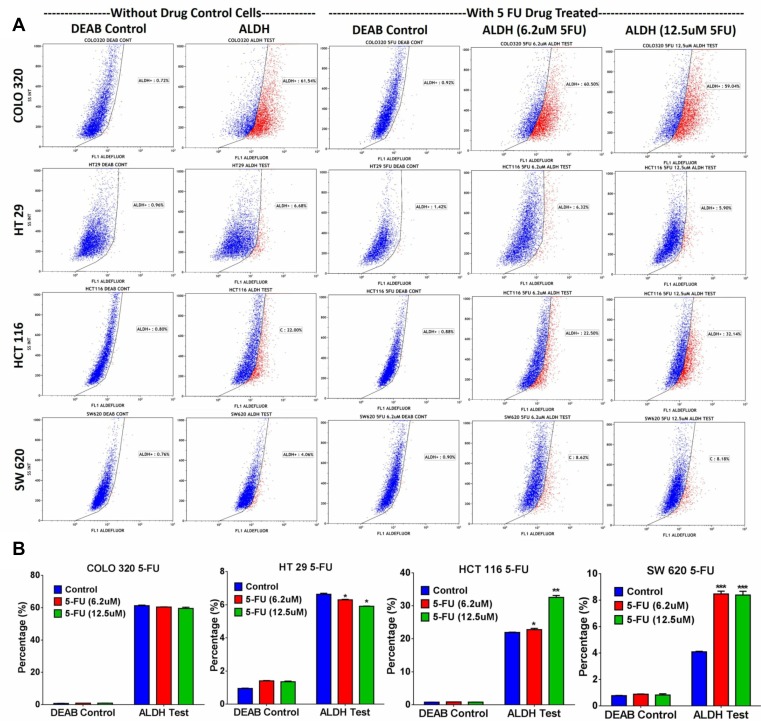
ALDH^+^ fraction is more resistant to 5-FU in multiple CRC models Drug sensitivity and enrichment analysis of ALDH^+^ populations in different CRC cell lines. (**A**) Different adenocarcinoma clones of CRC cell lines (COLO320, HT29, HCT116, and SW620) were treated with 5-FU for 5 days using different concentrations (6.2 µM and 12.5 µM), and subsequently the cells were stained with the Aldefluor assay to determine the frequencies of ALDH^+^ cells in 5-FU treated *vs* non-treated cells. Experiments were normalized with respective DEAB controls. (**B**) The two-tailed *t*-test was used to compare different treatment groups. ^*^p < 0.05; ^**^p < 0.005; ^***^p < 0.0005.

Microarray data revealed enrichment in DNA damage and oxidative stress response pathways in ALDH^+^ cells; thus, we hypothesized that ALDH^+^ cells exhibit enhanced resistance to 5-FU, a chemotherapeutic drug frequently used to treat patients with CRC. 5-FU treatment of SW403 cells led to a dose-dependent increase in the percentage of ALDH^+^ cells from 7% in controls to 24% at a concentration of 12.5 µM (Figure 3A and 3B). In addition, as shown in Figure 3C, the acridine orange/ethidium bromide (AO/EtBr) assay revealed a higher percentage of cell death (apoptosis and necrosis) in the ALDH1^−^ compared to the ALDH1^+^ fraction in the SW403 model in response to 5-FU (1.25 and 2.5 µM) treatment. To extrapolate our findings to additional colorectal cancer cell models, a panel of colorectal cancer cell lines: COLO320, HT29, HCT116, and SW620 were exposed to different concentrations of 5-FU for 5 days at a concentration of 6.2 and 12.5 µM and the percentages of ALDH^+^ cells were determined using the Aldefluor assay. As shown in Figure 4A and 4B, significant increases in the percentages of ALDH^+^ population were observed in the HCT116 (Control: 22%; 6.2 µM: 22.5%; 12.5 µM: 32%) and SW620 (Control: 4%; 6.2 µM: 8%; 12.5 µM: 8%) cells, whereas there were no significant changes in the percentage of ALDH^+^ population within the COLO320 and HT29 models following 5-FU treatment. Therefore, it is plausible that 5-FU treatment targets mainly the ALDH^−^ population, which leads to an increase in the ALDH^+^ fraction post-treatment.

### Inhibition of MAPK and FAK signalling pathways reduces the ALDH1^+^ cell fraction in the SW403 cell line

**Figure 5:**
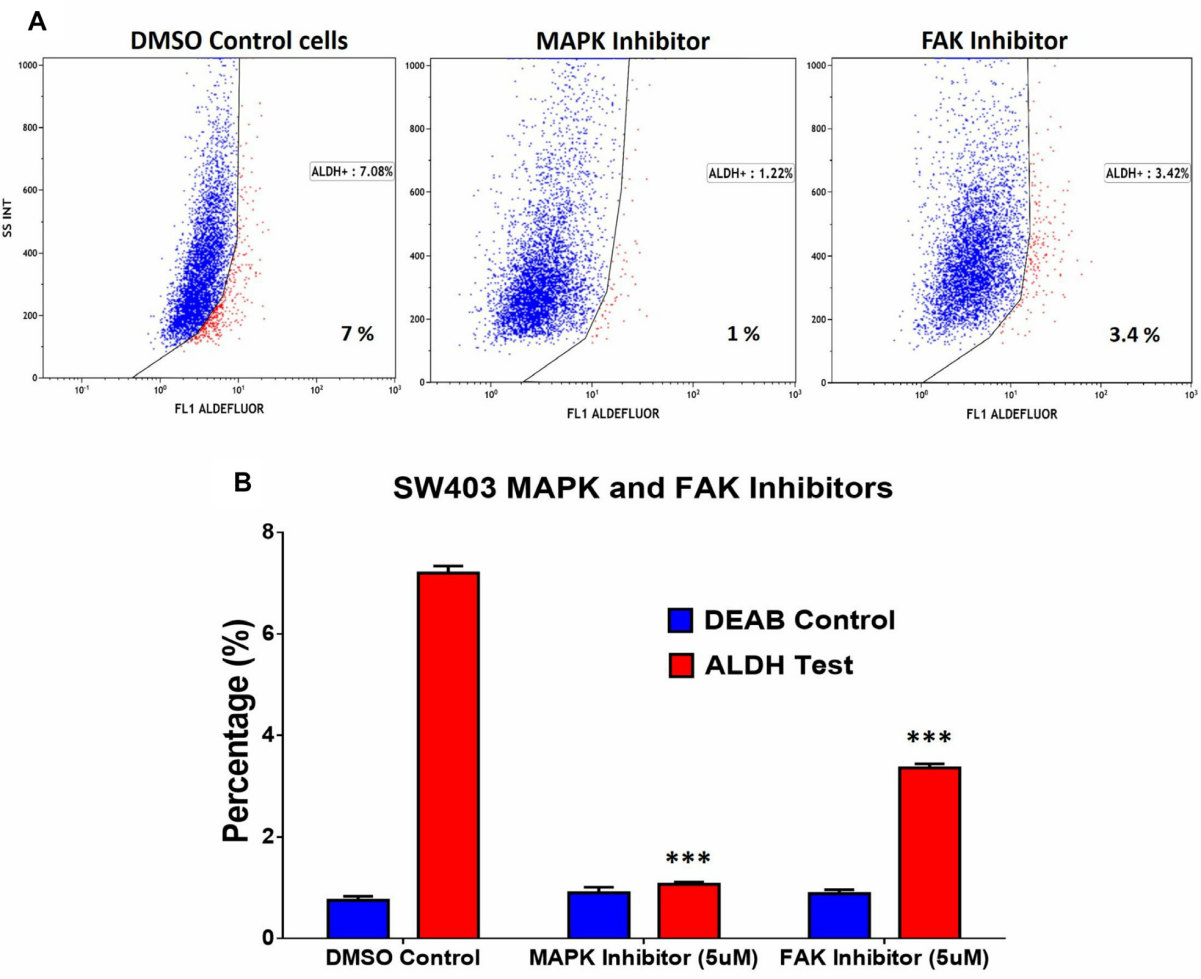
Pharmacological inhibition of MAPK and FAK significantly abrogates the ALDH^+^ population in the SW403 CRC model (**A**) Inhibition of MAPK ((5 μM, PD98059; Sigma; middle panel) or FAK ((5 μM, PF-573228; Sigma; right panel) for 5 days reduces the proportion of ALDH^+^ cells in the SW403 model. (**B**) Quantitative analysis of the frequencies of ALDH^+^ cells from (a). The two-tailed *t*-test was used to compare the treatment group with the respective control. ^***^p < 0.0005.

In addition to DNA damage and oxidative stress pathways, ALDH^+^ cell molecular signature revealed significant enrichment in genes within the MAPK and FAK signalling pathways (Figure 2B). Illustration of the FAK and MAPK pathways are shown with matched entities highlighted in Supplementary Figures 4 and 5, respectively. These data suggested a plausible role for MAPK and FAK signalling pathways in maintaining the ALDH^+^ population. SW403 cells were treated with MAPK (5 µM; PD98059) and FAK (5 µM; PF573228) small molecule inhibitors, and the ALDH1^+^ cell fraction was determined on day 5 post-treatment. As shown in Figure 5A and 5B, significant decreases in the ALDH1^+^ population were observed in both MAPK (1%) and FAK (3.4%) inhibitor-treated cells, compared to the dimethyl sulphoxide (DMSO) control (7%), suggesting a role for these two pathways in maintaining the CRC ALDH^+^ population. Whether MAPK/FAK inhibition promotes ALDH^+^ cell death or reduced ALDH expression remains to be investigated.

### ALDH1^+^ cells are more resistant to oxidative stress compared to ALDH1^−^ cells

**Figure 6:**
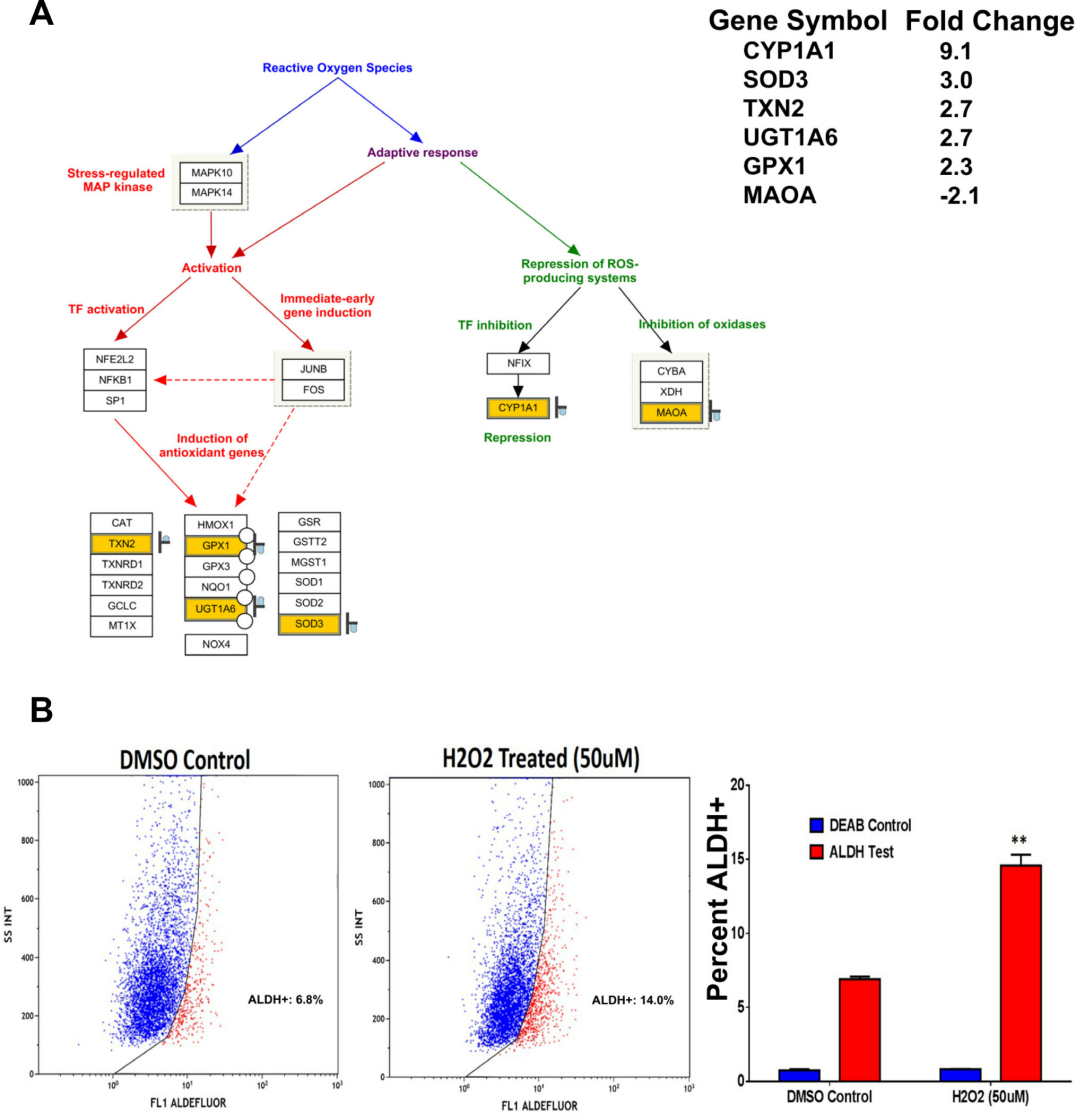
ALDH^+^ cells exhibit enhanced resistance to oxidative stress (**A**) Illustration of the oxidative stress pathway with matched entities highlighted and fold change in ALDH^+^ vs ALDH^-^ indicated. (**B**) Significant increase in the frequencies of ALDH^+^ positive population in the SW403 cell line was observed post-exposure to H_2_O_2_ (50 µM). Quantitate analysis of the frequencies of ALDH^+^ cells is shown in the right panel. Data are presented as the means ± S.D. Two-tailed *t*-test was used to compare treatment groups. ^**^p < 0.005.

Hydrogen peroxide (H_2_O_2_) is a non-radical reactive oxygen species (ROS) that can activate nuclear transcription factors, such as NF-κB, p53, and AP-1 leading to the induction of pro-apoptotic or inhibitor of survival proteins [21]. The oxidative stress pathway is illustrated with matched entities highlighted in Figure 6A. As we found that the oxidative stress pathway was one of the top enriched pathways in ALDH1^+^ cells (Figure 2B), we examined the effect of ROS on the ALDH^+^ cell fraction in SW403 cells following exposure to H_2_O_2_ (50 µM) for 5 days. Our data revealed a significant increase in the ALDH^+^ fraction (14% ALDH^+^) compared to the control cells (7% ALDH^+^), suggesting that ALDH^+^ cells are more capable of surviving ROS-mediated oxidative stress (Figure 6B).

### ALDH1 expression is a possible prognostic marker for CRC

**Figure 7:**
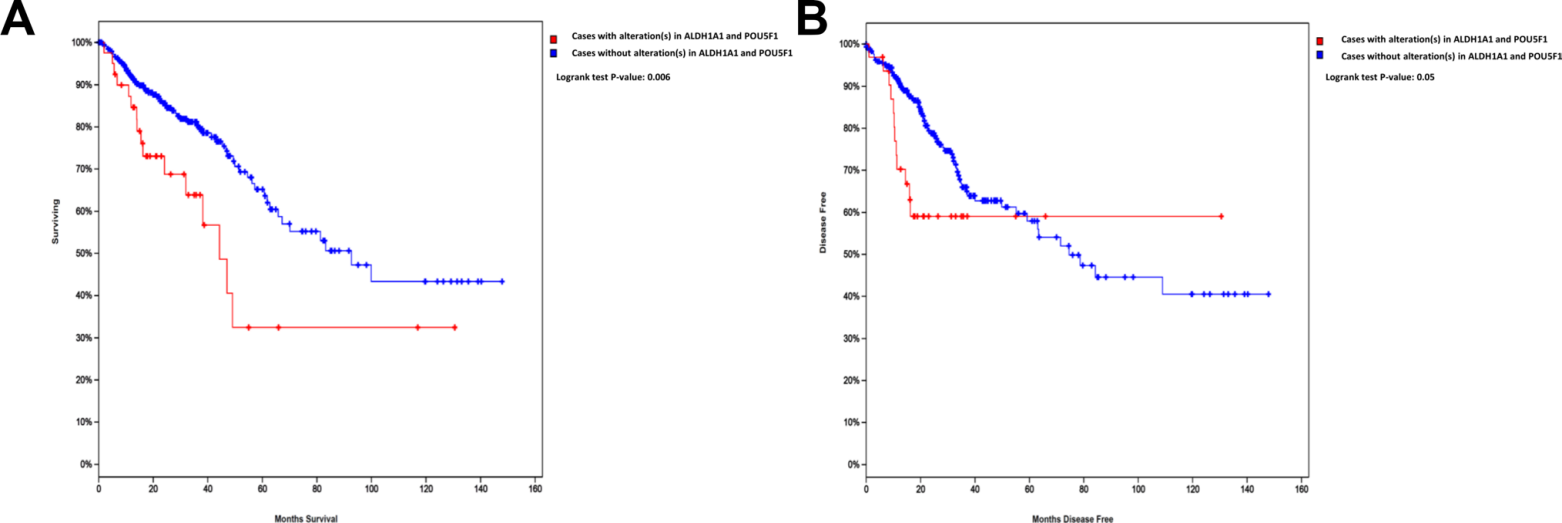
Altered expression of *ALDH1A1* or *POU5F1* is associated with poor OS and DFS in CRC Kaplan–Meier curves illustrate the duration of OS (**A**) or DFS (**B**) according to the expression of *ALDH1A1* and *POU5F1* in a cohort of 462 patients (for OS) or 420 patients (for DFS) from the TCGA colorectal cancer dataset. Using log-rank analysis; the expression of *ALDH1A1* or/and *POU5F1* was associated with poor OS (*p* = 0.006) and DFS (*p* = 0.05).

To assess the clinical relevance of our findings, we examined the expression of *ALDH1A1* and *POU5F1* in a cohort of 462 patients for overall survival (OS) or 420 patients for disease-free survival (DFS), obtained from the Cancer Genome Atlas CRC data set [22]. As shown in Figure 7, a strong association between elevated expression of *ALDH1A1* or/and *POU5F1* and poor OS (*p* = 0.006) and poor DFS (*p* = 0.05) was observed.

## DISCUSSION

CSCs have been identified in a number of solid tumours including breast, colon, glioma, liver, lung, melanoma, ovarian, pancreatic, and prostate cancers. However, their biological relevance and functions within the tumour microenvironment remains under investigation and debate [8, 23]. The true identify of CSCs is also still uncertain, although a number of cellular proteins have been suggested as potential markers for CSCs [24]. Among these, CD133 (PROM1), and LGR5 have been suggested as potential markers for CRC CSCs [25, 26]. Notably, the frequencies of CD133^+^ or LGR5^+^ in CRC can reach >24%, which may question their specificity for identifying CSCs. ALDH, alternatively, has been suggested as a potential marker for normal colon as well as CRC SCs [27]. ALDH^+^ cells are few in number and limited to the normal crypt bottom, the expected location of SCs. An *APC* gene mutation that leads to epithelial development of adenoma is associated with increased number of ALDH1^+^ cells and their distribution beyond the crypt. Furthermore, ALDH1^+^ cells isolated from patients with CRC readily generated xenograft tumours with as low as 25 cells injected *in vivo*, whereas ALDH1^−^ cells did not form tumours [27].

In the present study, we found a distinct molecular phenotype of ALDH^+^ cells, which suggests a number of biological characteristics relevant to understanding the biology of CRC and its response to therapy. Pathway analysis on the up-regulated genes in ALDH^+^ cells revealed significant enrichment in multiple signalling pathways including FAK, MAPK, DNA damage response, cell cycle, oxidative stress, and Wnt and pluripotency pathways. Concordant with the gene expression data, functional studies demonstrated a pivotal role for FAK and MAK signalling in the maintenance of ALDH^+^ cells. Some recent studies corroborate our findings. Blaj *et al.* demonstrated strong intra-tumoural heterogeneity with respect to activation of MAPK in CRC, with high MAPK activity restricted to the less-differentiated tumour cells located at the tumour leading edge [28]. Additionally, elevated expression of genes associated with MAPK and FAK signalling have been linked to epithelial-to-mesenchymal transition in CRC [29–32]. FAK is overexpressed and is activated in numerous advanced-stage solid tumours. In addition, FAK has been described as an important pathway in CSC self-renewal and cancer metastasis through both kinase-dependent and kinase-independent mechanisms [31]. In acute myeloid leukaemia, the FAK pathway regulates the expression of a number of cytokines (interleukin 6 (IL 6), IL 8, stromal cell-derived factor 1, and angiopoietin 1), which are crucial for CSCs maintenance [33]. In animal models, small-molecule FAK inhibitors reduce tumour angiogenesis and FAK inhibitors are being developed for a possible role in cancer therapy [34].

We observed that ALDH^+^ cells were more resistant to 5-FU treatment and ROS exposure than ALDH^−^ cells in several CRC cell models: SW403, HCT116, and SW620, suggesting a possible role in mediating drug resistance. In other human cancer models, CD133 positive CSCs were reported to contribute to glioma radioresistance through preferential activation of the DNA damage checkpoint and an increase in DNA repair ability [35]. Similarly, Lim *et al.* confirmed that glioma CSCs play an important role in radioresistance through initiation of DNA damage checkpoint proteins including ATM, SMC1, Chk1, Chk2, and p53 and increased DNA repair [36]. In our study, we observed ALDH^+^ cells to highly express a pluripotency gene (*POU5F1*, also referred to as *OCT4*) and superoxide dismutase 3 (*SOD3*), suggesting a role for these two genes in driving stemness and promoting cell survival under various stress conditions in ALDH^+^ cells. Concordant with our data, Chiou *et al.* reported that co-expression of POU5F1 and Nanog could enhance the malignancy of lung adenocarcinoma through induction of CSC-like properties and epithelial mesenchymal transition [37]. Similarly, Kumar and colleagues showed that POU5F1 was able to promote dedifferentiation of melanoma cells into CSC-like cells [38]. Taken together, these findings clarify that POU5F1 plays a crucial role in maintaining the CSC phenotype in multiple human cancer types.

The 5-year survival rate for patients with CRC having a localized tumour is approximately 90%, which is reduced to 70% for patients presenting with regional disease, and to 12% for those with metastases [39]. Therefore, a significant number of patients with CRC do not appear to benefit from standard chemotherapy, in particular in metastatic disease. Our *in vitro* data suggest that the presence of ALDH^+^ cells might predict the response of CRC to therapy. A number of studies have examined the relevance of *ALDH1A1* as a biomarker in CRC. Nuclear expression of *ALDH1A1* in a small subpopulation of patients was associated with shortened survival [40]. In a recent retrospective study, immunohistochemical expression of ALDH1 in epithelial cells was associated with poor prognosis, whereas its expression in stromal cells was associated with good prognosis in CRC [41]. In another study, high ALDH1 expression was found as an independent prognostic factor associated with the 5-year OS and DFS and correlated with the tumor stage, lymph node status, and tumor differentiation [42]. These findings corroborate our findings of the association of *ALDH1A1* expression with poor prognosis of the disease. Thus, our data revealed multiple enriched pathways within ALDH^+^ CRC cells that could potentially be targeted to eliminate the CSC population within the tumour, with the aim of treatment of disseminated disease and prevention of recurrences [9]. While the gene expression profiling and pathway data presented in current study were generated using the non-metastatic SW403 model, additional testing is needed to validate whether similar molecular signature also exists in metastatic colorectal cancer cell lines.

## MATERIALS AND METHODS

### Cells lines and tissue culture

The human colorectal cancer cell lines (SW403, COLO320, HT29, HCT116, and SW620) were obtained from American Type Culture Collection (ATCC, Manassas, VA, USA). Cells were maintained in Dulbecco’s modified Eagle’s medium supplemented with 10% foetal bovine serum (Gibco-Invitrogen, Waltham, MA, USA) and 100 mg/l penicillin/streptomycin. All cells were maintained in a 37° C incubator with humidified 5% CO_2_.

### Immunophenotyping by flow cytometry (FACS)

Immunophenotypic analysis was performed in accordance with our previously published protocols [12]. In brief, SW403 cells were harvested using 0.05% trypsin-ethylenediaminetetraacetic acid and were washed twice in ice-cold phosphate buffered saline (PBS) supplemented with 0.5% bovine serum albumin and resuspended at 10^6^ cells/ml. Then, 10 μl of FITC-conjugated mouse anti-human CD24 and CD90, PE-conjugated mouse anti-human CD44, CD29, and EpCAM, or APC-conjugated mouse anti-human CD133 and LGR5 antibodies (from BD Biosciences, San Jose, CA, USA, and Miltenyi Biotec, Bergisch-Gladbach, Germany) was added to 100 μl of cell suspension (10^5^ cells). Negative control staining was performed using FITC, PE, or APC-conjugated mouse IgG1 isotype control antibodies, respectively. Cells were incubated for 30 min at 4° C in the dark, washed with PBS, resuspended in 500 μl of PBS, and analysed using a Navios flow cytometer (Beckman Coulter, Brea, CA, USA). Living cells were gated in a dot plot of forward vs. side scatter signals obtained on linear scale. At least 5000-gated events were acquired on a log fluorescence scale. Data were analysed using Kaluza software (1.2 version, Beckman Coulter).

### Aldefluor assay and ALDH +/− cell sorting

Aldefluor assay was performed in accordance with our previously published protocols [43]. The Aldefluor kit (Stem Cell Technologies, Vancouver, BC, Canada) was used to determine the percentage of cells with high aldehyde dehydrogenase (ALDH) enzymatic activity. Briefly, 10^6^ cells were resuspended in Aldefluor assay buffer containing ALDH substrate as recommended by the manufacturer. As a negative control for all samples, an aliquot of ‘Aldefluor-exposed’ cells was immediately quenched using the ALDH inhibitor DEAB. After 30 min of incubation at 37° C, the cells were centrifuged and resuspended in 500 μl Aldefluor buffer and analysed using a Navios flow cytometer. Aldefluor staining was detected within the green fluorescence channel FL1. Samples treated with the inhibitor DEAB (+DEAB) were used as controls to establish the gates defining the ALDH^+^ region. DMSO control cells were used as an experimental control to compare drug or inhibitor treated cells, respectively. Kaluza software (1.2 version) was used to analyse the data. ALDH^+/−^ cells were collected using a MoFlo Astrios cell sorter (Beckman Coulter). The collected cells were directly used for further experiments.

### Small molecule inhibitor experiments

Unsorted SW403 cells were exposed for 5 days to MAPK (5 µM; PD98059) and FAK (5 µM; PF573228) inhibitors, whereas oxidative stress was induced using H_2_O_2_ (50 µM). On day five, the cells were washed and the percentage of ALDH^+^ cells were analysed by the Aldefluor kit.

### Gene expression microarray

RNA isolation and gene expression analyses were carried out as described in our previously published manuscripts [44, 45]. In brief, RNA was isolated using the Total Tissue RNA Purification Kit from Norgen-Biotek Corp. (Thorold, ON, Canada) and was quantified using NanoDrop 2000 (Thermo Scientific, Wilmington, DE, USA). Total RNA was labelled and then hybridized to the Agilent Human SurePrint G3 Human GE 8 × 60 k mRNA microarray chip (Agilent Technologies, Santa Clara, CA, USA). All microarray experiments were conducted at the Microarray Core Facility (Stem Cell Unit, Department of Anatomy, King Saud University College of Medicine). Data were subsequently normalized and analysed using GeneSpring 13.0 software (Agilent Technologies). Pathway analyses were conducted using the Single Experiment Pathway analysis feature in Gene Spring 13.0 (Agilent Technologies). Twofold cut-off with *p* < 0.02 was used.

### Gene validation using qRT-PCR

Gene expression levels were validated in sorted SW403 ALDH^+^ and ALDH^−^ cells using an RT-PCR assay as described previously [44]. In brief, SYBR Green-based qRT-PCR was performed using an Applied Biosystems ViiA 7 Detection system (Foster City, CA, USA). Then, 500 ng of total RNA was reverse transcribed using a High Capacity cDNA Reverse Transcript Kit (Part No: 4368814; ABI) according to the manufacturer’s protocol. Relative levels of mRNA were determined from cDNA using quantitative real-time PCR (Applied Biosystems ViiA 7 Systems). Primer sequences used in the current study are listed in Table 1. The expression level was calculated relative to *GAPDH* as a control.

**Table 1:** List of SYBR green primers used in current study

No	Name	Sequence
1	*GAPDH*	5¢ CTGGTAAAGTGGATATTGTTGCCAT
		5¢ TGGAATCATATTGGAACATGTAAACC
2	*CDC25B*	F 5¢ GCTCTCAGTCCAGCAGGC
		R 5¢ ACTCTTTGGGGTTTCGCTGC
3	*CCND3*	5¢ GCCTCCTACTTCCAGTGCG
		5¢ CCTCACATACCTCCAGCATCC
4	*ATM*	5¢ TGCGTGGCTAACGGAGAAAA
		5¢ ATCACTGTCACTGCACTCGG
5	*TP53AIP1*	5¢ CTGGCTGGGTTTCAGATCCC
		5¢ CAGGCAAGCTCTTACTGCAC
6	*SOD3*	5¢ CTGGAAAGGTGCCCGACTC
		5¢ CTTGGCGTACATGTCTCGGA
7	*CYP1A1*	5¢ CCCACAGCACAACAAGAGAC
		5¢ GGGTGAGAAACCGTTCAGGT
8	*POU5F1*	5¢ TCTTCAGGAGATATGCAAAGCAGA
		5¢ GATCTGCTGCAGTGTGGGT

### Measurement of cell proliferation and clonogenic assay

The proliferation of sorted ALDH^+^ and ALDH^−^ cells was determined using an Alamar Blue assay as previously described [4]. Briefly, 3000 cells were cultured in a 96-well plate and proliferation was measured at the indicated time points by adding 10% volume Alamar Blue assay reagent and measuring absorbance at 570 λ. The colony forming ability of both ALDH^+^ and ALDH^−^ cells was determined using a clonogenic assay as previously described [44]. Briefly, both SW403 SW403 ALDH^+^ and ALDH^−^ cells were seeded in 12-well plates in different serial dilution (1:2 to 1:16). Initial seeding density was 0.015 × 10^6^ cells per well, and plates were incubated at 37° C under 5% CO_2_ for 10 days. The plates were then washed and stained with Diff-Quik stain set (Siemens, Malvern, PA, USA), scanned, and the number of colonies was counted using Image-Pro Plus software (Media Cybernetics). The experiment was performed twice in duplicate. The fraction of surviving cells was estimated by comparing the number of colonies formed in ALDH^+^ to that in the ALDH^−^ cells.

### Measurement of apoptosis

A fluorescence-based apoptosis assay was employed in cells following exposure to different concentration of 5-Fluorouracil (2.5 to 1.25 µM), using the AO/EtBr staining method as previously described [44]. Briefly, cells were stained with dual fluorescent staining solution (1.0 µl) containing 100 µg/ml AO and 100 µg/ml EtBr (AO/EB, Sigma, St. Louis, MO, USA). Cells were gently mixed with AO/EtBr (1:100) dye solution for 1 min; afterwards, the cells were observed and photographed under a Nikon Eclipse Ti fluorescence microscope (Nikon, Tokyo, Japan). Cells cultured without drug treatment were considered control.

### Statistical analysis

Statistical analyses and graphing were performed using Microsoft excel 2010 and GraphPad Prism 6.0 software (GraphPad, San Diego, CA, USA). *P*-values were calculated using a two-tailed *t*-test.

## SUPPLEMENTARY MATERIALS FIGURES AND TABLE





## Abbreviations

CSCs: cancer stem cells
CRC: colorectal cancer
OS: overall survival
DFS: disease-free survival
ALDH1: aldehyde dehydrogenase 1
DEAB: diethylaminobenzaldehyde
qRT-PCR: quantitative reverse transcription polymerase chain reaction
DMSO: dimethyl sulphoxide
PBS: phosphate buffered saline
