# Supplementary material for: Molecular profiling of ALDH1+ colorectal cancer stem cells reveals preferential activation of MAPK, FAK, and oxidative stress pro-survival signalling pathways
Source: Oncotarget. 2018 Feb 5;9(17):13551–64. doi: 10.18632/oncotarget.24420 (PMC5862598; doi:10.18632/oncotarget.24420)
Supplement: Supplementary file 1 [file oncotarget-09-13551-s001.pdf]

Molecular profiling of ALDH1+ colorectal cancer stem cells reveals preferential activation of MAPK, FAK, and oxidative stress pro-survival signalling pathways

SUPPLEMENTARY MATERIALS

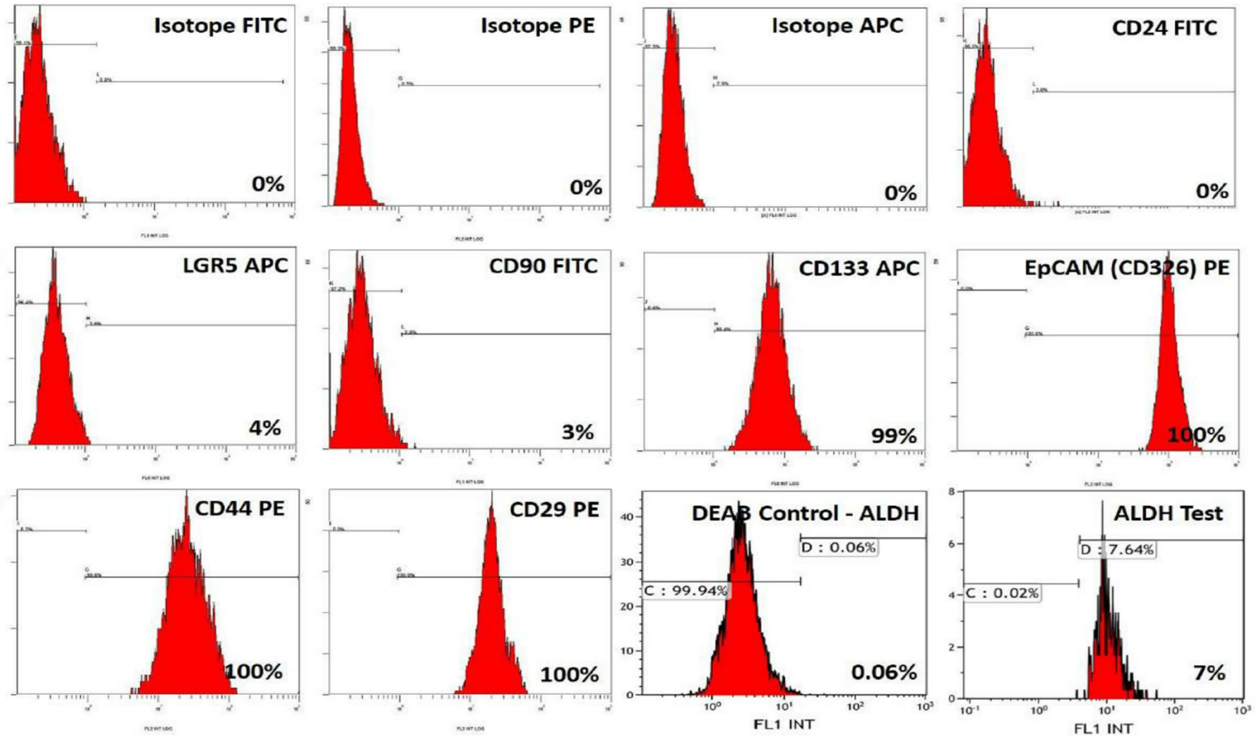

Supplementary Figure 1: Flow Cytometry analysis with cancer stem cell associated markers in SW403 CRC model.

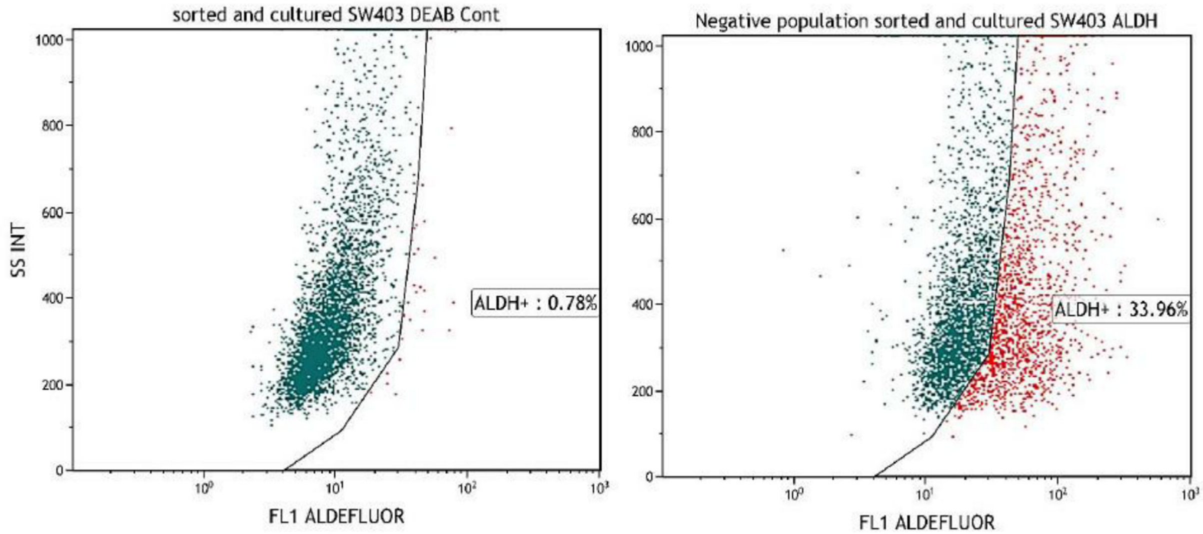

Supplementary Figure 2: Expression of ALDH in cultured sorted ALDH– SW403 cells. Sw403 cells were sorted based on ALDH expression and subsequently they were culture. The percentage of ALDH+ was subsequently assessed in cultured cells using the A ldefluor assay.

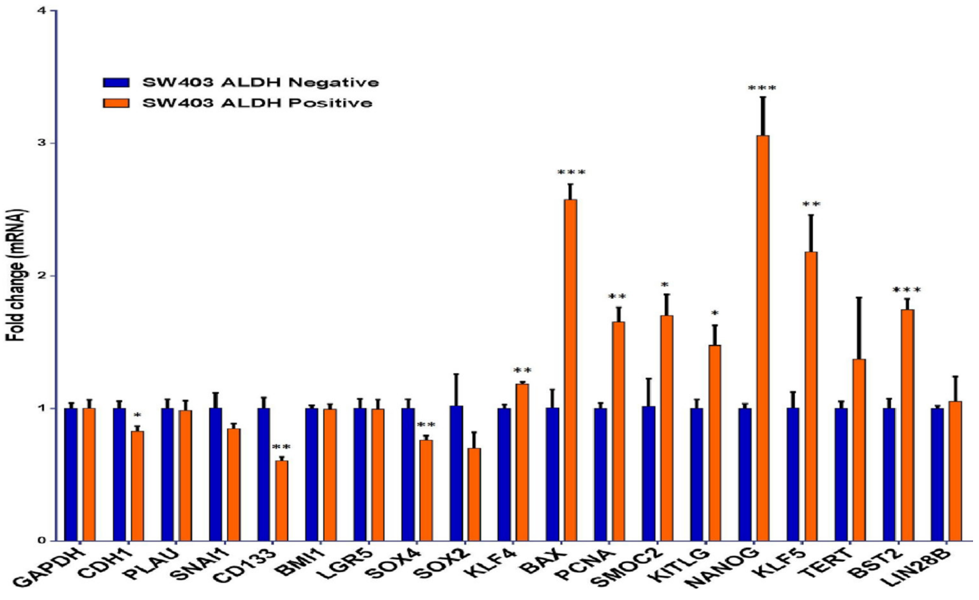

Supplementary Figure 3: Expression of cancer stem cell associated gene markers in ALDH+ and ALDH– SW403 CRC model.

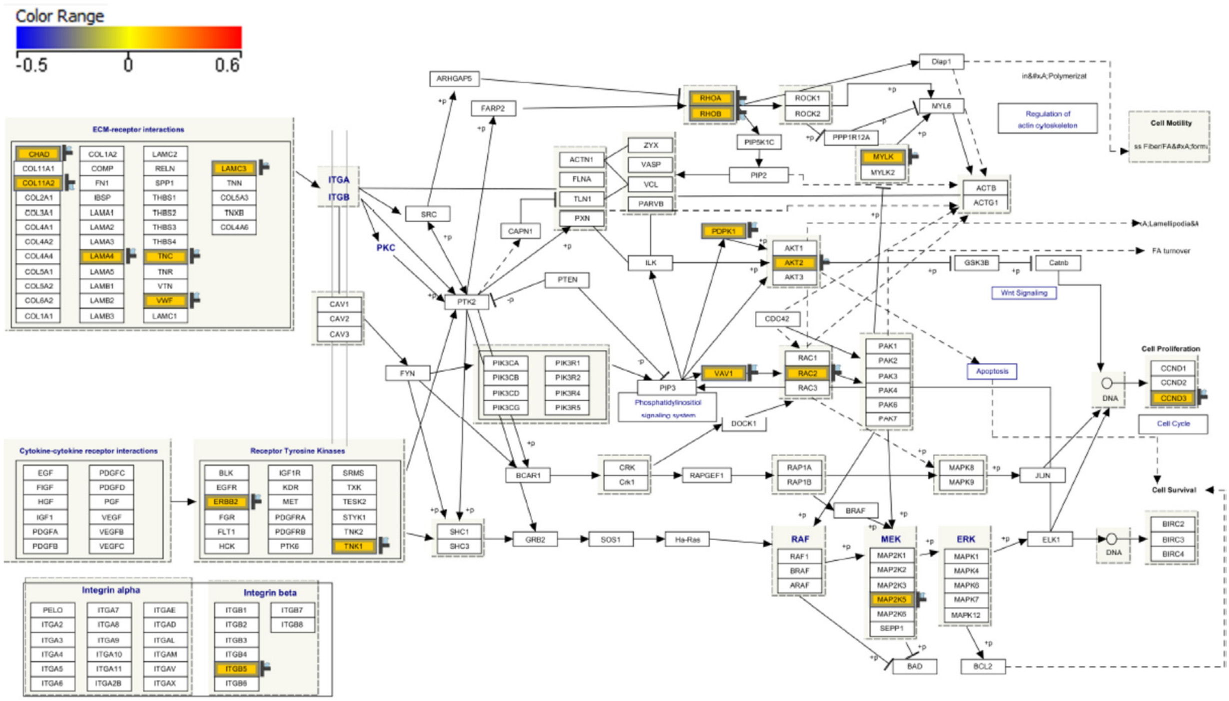

Supplementary Figure 4: Illustration of the Focal Adhesion pathway based on microarray data for ALDH+ vs ALDH– SW403 cells with matched entities highlighted.

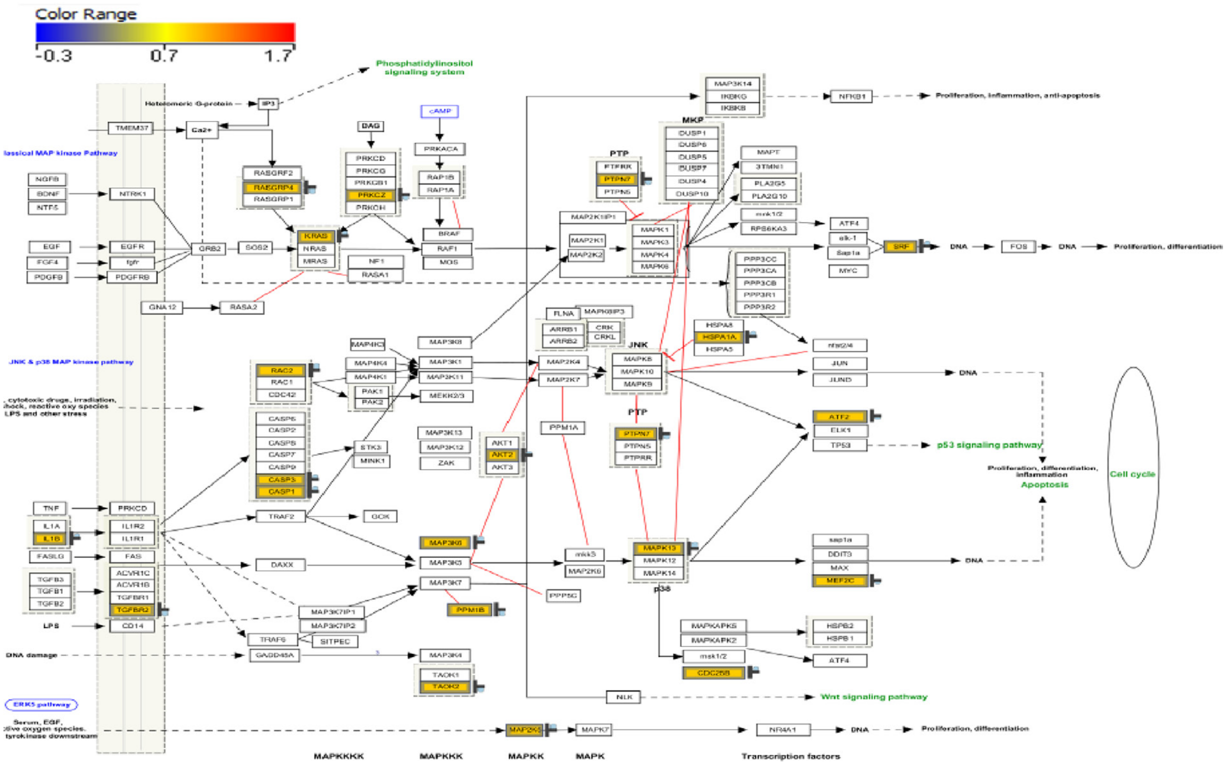

Supplementary Figure 5: Illustration of the MAPK signaling pathway based on microarray data for ALDH+ vs ALDH– SW403 cells with matched entities highlighted.

Supplementary Table 1: List of differentially expressed genes (2.0 FC, *p* < 0.05) in ALDH+ vs ALDH- SW403 CRC cells. See Supplementary_Table_1
